# Mandatory substance use treatment for justice-involved persons in Germany: a systematic review of reoffending, treatment and the recurrence of substance use outcomes

**DOI:** 10.3389/fpsyt.2023.1217561

**Published:** 2024-02-05

**Authors:** Jack Tomlin, Esther Meise, Juliane Wegner, Birgit Völlm

**Affiliations:** ^1^School of Law and Criminology, University of Greenwich, London, United Kingdom; ^2^Department of Forensic Psychiatry, University Medicine, Rostock, Germany; ^3^Institut für Medienforschung, University of Rostock, Rostock, Germany

**Keywords:** forensic mental health, substance use disorder, mandatory treatment, Germany, reoffending

## Abstract

Many jurisdictions implement mandatory substance use treatment for justice-involved persons. Germany is one such country; however, debates about the appropriateness and effectiveness of this disposal abound. Very little attention has been paid in the international literature to patients receiving mandatory treatment in Germany. This systematic review synthesises research on patients receiving substance use treatment in forensic hospitals under §64 of the German Penal Code with regard to three primary outcomes: treatment completion, reoffending, and the recurrence of substance use. Forty-five publications reporting on 36 studies were reviewed; publication dates ranged from 1988 to 2023. On average, 47% of patients did not successfully complete treatment, compared to 45% who did. Average follow-up reconviction rates were higher than in mentally ill and general offender populations as reported elsewhere. Approximately half of all patients reused substances during treatment. Suggestions for future research, including a focus on strength- and recovery-based indicators, and harmonising routine outcomes measurements, are given.

## 1 Introduction

### 1.1 Substance use and treatment in the criminal justice system

Substance misuse is strongly linked to criminal behaviour (1). This association is described as a ‘dynamic relationship’, as the literature recognises that the links between drug-use and criminal behaviour are multi-directional (2, 3). A history of substance misuse has been identified in the literature as a strong predictor of violence and recidivism in both the general offender population (4) and for offenders with mental health disorders (5, 6). In a systematic review of 18,388 newly admitted inmates across 24 studies, the authors found that approximately 30% of men and 51% of women had a drug misuse disorder, and a quarter of both sexes had an alcohol misuse disorder (7).

Given the prevalence of substance use disorders in the criminal justice settings (CJS), various efforts aim to treat or divert people with substance use disorders away from traditional criminal justice disposals (8). Alternative approaches include the use of drug courts, diversion schemes, mandatory or voluntary treatment programmes, pharmacological and psychosocial interventions, provided across a range of settings including secure hospitals, prisons, and various forms of support or monitoring in the community (1). A comprehensive meta-analysis of 28 studies by Holloway et al. (9) concluded that the likelihood of reoffending by individuals assigned to some form of treatment was 41% lower than controls not receiving such an intervention.

Evidence as to the effectiveness of different types of interventions is mixed (1). For instance, although some studies suggest that for non-substance abusing offenders, community sentences lead to lower reoffending rates than penal sentences (3), interventions targeting offenders with substance use disorders in the community are less effective than intensive programmes in secure environments (9). Research indicates that programmes combining pharmacological and psychosocial elements are more effective than either delivered individually, and that therapeutic communities, which embody a secure holistic treatment approach, receive the most empirical support (1). A review of 43 trials using non-pharmacological interventions for criminal justice-involved persons found that on average these led to a significant reduction in re-incarceration and a near significant reduction in substance use, but not in re-arrests; therapeutic communities were the only modality that led to significant reductions in re-arrests (10). Further, interventions are differentially effective; non-white, young men are more likely to benefit than other groups (9).

### 1.2 Mandatory treatment and quasi-compulsory treatment

Mandatory treatment is defined as *‘any form of drug treatment that is ordered, motivated, or supervised by the criminal justice system’* [(11), 2]. In Germany, Austria and Switzerland, treatment can be ordered without requiring an individual’s consent.

The evidence is mixed on the question of whether legal compulsion as compared to voluntary programmes is associated with better outcomes (12). A review of the effectiveness of mandatory treatment interventions for people with substance use disorders found nine relevant studies (13). Of these, three studies reported no difference between mandatory treatment and controls on substance use outcomes, two studies found ‘equivocal’ links to positive substance use outcomes but there were no control groups, two studies reported positive outcomes, and two studies observed negative impacts on criminal recidivism (i.e., higher reoffending rates).

One of the reasons for heterogeneity in outcomes is the wide variation in programme intensity, modality, setting, and patient population. A review of coerced treatment programmes for substance using offenders identified programme- and individual-level factors that were associated with more successful outcomes (14). At the programme-level, these included, among other things, programmes that: lasted longer than 90 days, had compliance measures to enforce requirements, incorporated motivation and reinforcement techniques, ensured a continuum of care across the criminal justice system, and employed staff who had accreditation and training. At the individual-level, the authors report that greater motivation, treatment readiness, higher levels of religion and faith, and lower baseline stress levels were associated with better substance use and offending outcomes. Co-occurring mental disorders were linked with higher recidivism rates, and criminal thinking styles with poor engagement in treatment.

### 1.3 Mandatory treatment for offenders with substance use disorders in Germany

The German criminal justice system provides for a form of mandatory treatment for offenders whose convictions are considered by a court to relate to substance use. §64 of the German Penal Code (Strafsgesetzbuch; StGB) stipulates that where an offence can be linked to a substance use disorder or is committed in a state of intoxication, there is risk of future substance use-related offending, and there are reasonable prospects that treatment might be successful, an individual can be ordered into a forensic mental health hospital specialising in the treatment of substance use disorders. These hospitals are secure forensic-psychiatric settings, the vast majority of which have secure perimeters. Some such hospitals specialise in treating individuals detained under §64 StGB while others also treat offender patients with other disorders having been committed under a hospital order with unlimited duration by the courts. There is no distinction of hospitals by security levels in Germany. The vast majority of forensic-psychiatric hospitals offer care at all levels of security but differentiate security levels internally within the same institution. Treatment models vary across these sites, though typically multidisciplinary, holistic treatment programmes involving a range of psychological, social, occupational, pharmacological, and substitution treatments are offered (15).

The use of §64 StGB is independent of criminal responsibility; in practice about 60% of the individuals detained have full, and most of the remainder diminished, criminal responsibility. Both these groups receive a parallel prison sentence and could be returned to prison to serve this sentence if treatment does not appear to be successful. Detention under §64 StGB is ordered for a period of 2 years, but can be extended by up to 2/3 of the length of the prison sentence ordered in parallel (16). In case of long prison sentences, individuals will be ordered to serve part of the prison sentence prior to mandatory treatment in order to avoid them having to return to prison after successful completion of the treatment (15). Placement is reviewed every 6 months. As is common internationally, most patients are men (87.9%); the average age is 37 years (17, 18). Average treatment length is 3.5 years (15). There were 4,300 §64 German Penal Code patients in Germany in 2019, a marked increase from 1,657 in 1999 (18). The reasons for this increase are not fully understood. However, a number of factors have been identified accounting for this observation: 1. There has been an increase in the average lengths of parallel prison sentences (by 9 months over a period of 20 years) due to an increase in severe (violent) offences. 2. As a consequence, average duration of treatment has also increased (by 6 months). 3. The characteristics of patients have changed, in particular, the percentage of patients admitted with full criminal responsibility has increased due to a wider interpretation of the entry criteria by the courts. This has led some to argue that there are fewer ill and a greater number of dissocial patients with limited treatment motivation and poor prognosis now residing in forensic psychiatric care. Some have suggested that the possibility of early release provides a false incentive for this group (for a summary of the discussions see Bundesministerium für Justiz, 2022).

Approximately half of all patients do not successfully complete treatment (15). This has raised questions about whether the right individuals are being selected for mandatory treatment in the first instance and led to calls, including from the German Association for Psychiatry, Psychotherapy and Psychosomatics (DGPPN), to significantly reform the admission criteria for treatment under this legal provision, including making such treatment voluntary (19).

Knowledge of the outcomes for the §64 German Penal Code treatment population and the factors predictive or associated with these outcomes in the international (English language) literature is sparse. A recent study found that patients successfully completing treatment took significantly longer to reoffend (*N* = 110, *M* = 46 months) than those transferred to prison (*N* = 151, *M* = 25 months) (20). A review by Fries et al. (21) of nine papers published between 1999 and 2009 in German reported that young age, history of offending, lack of educational and vocational attainment, and comorbid personality disorders were the most robust predictors of treatment completion. A more recent review of 16 studies published after 1999 in German looking at predictors of treatment completion found that age, past criminal convictions, comorbid personality disorders, use of substances other than alcohol, educational and vocational attainment, withdrawal from previous substitution programmes, and needs mostly identified as substance-related instead of criminogenic, are the strongest predictors (22). A second review conducted at the same time by the same authors address predictors of reoffending after discharge (23). The authors report mixed findings but suggest that patient motivation, living circumstances and resources, and offending history were the most reliable predictors.

### 1.4 Aims and rationale

The present review aimed to synthesise the literature describing studies of the mandatory substance use treatment patient population in Germany (§64 StGB) in relation to three primary outcomes: reconviction/reoffending, treatment completion and the recurrence of substance use. It summarises the reported prevalence rates for these outcomes and describes the evidence-base for factors/predictor variables associated with these outcomes. This review extends the findings of the previous reviews by additionally reporting on reconviction/reoffending during treatment and the recurrence of substance use during treatment and after discharge. It also is the first English-language review of this literature. The German system is worthy of study as the largest European jurisdiction to have mandatory substance use treatment, is currently reviewing laws relating to this, and little has been published on this system in the international literature.

## 2 Materials and methods

The review protocol was published on PROSPERO [CRD42020148726]. The following search terms were used: [(psych* or mental*) AND (forensic* or secur* or crime* or criminal* or offend* or offence* or arrest* or prisoner* or inmate* or incarcerate* or quasi-compulsory or qct) AND (substance* or drug* or alcohol* or misuse* or addict*) AND (outcome* or predict* or associate*)]. The following databases were included in the review: PubMed; Web of Science; Embase; PsycINFO; MEDLINE; Cochrane Library; Google scholar (first 10 pages); Google search (first 10 pages); and the references of included articles were scanned to identify any further studies.

The following search parameters were set: the time limit included all records available for each database. Articles in English and German were included. Only studies of German services were included. An initial search was conducted in October 2019 and an updated search was conducted in November 2023.

### 2.1 PICOS and inclusion criteria

#### 2.1.1 Population

In-patients in forensic psychiatric care settings that have a primary diagnosis of substance or alcohol misuse and have been mandated treatment for this in Germany under §64 StGB. Individuals found to have committed an offence with full, partial, or absent responsibility were included. Both male and female patients over 18 years old were included. Patients that were released from forensic settings into the community or other services (general psychiatric, penal settings, out-patient settings) were included for follow-up (outcome) data.

#### 2.1.2 Intervention

Placement within a secure forensic setting under §64 StGB for the purposes of receiving treatment for substance or alcohol use diagnosis (not as a secondary diagnosis). People in jail/prison who happen to also receive an intervention for substance misuse were not included.

#### 2.1.3 Comparison

Not applicable.

#### 2.1.4 Outcomes

Primary outcomes were treatment completion, reoffending, and the recurrence of substance use. To support comparison of findings across studies, we defined *successful treatment* as: patients discharged into probation and those who reached the end of the legally-defined maximum length of treatment and were discharged; *unsuccessful treatment* included those patients for whom study authors reported there was ‘treatment failure’, were transferred to prison, or treatment was for another reason ended prematurely; and *other outcomes* as death or still being in treatment at time of data collection.

*Reoffending* was defined as (1) officially recorded reconvictions in the ‘Bundeszentralregister’ (Federal Central Register) after discharge from treatment, (2) officially recorded reconvictions in the Federal Central Register during treatment, (3) self-reported reoffending after treatment discharge, and (4) self-reported reoffending during treatment. Findings are described by category. Types of reoffences/reconvictions are not discussed in-depth as most studies reported any type of offending and did not investigate offence types (e.g., violent, sexual, property).

The *recurrence of substance use* is defined as any official, hospital, or self-reported record of substance use (illicit or licit, excluding caffeine and nicotine) in the study observation period. Very few studies distinguished between types of substances to a level of specificity greater than ‘alcohol’ or ‘other illicit substances’. Therefore, the recurrence of substance use is not reported according to type of substance unless where the distinction between ‘alcohol’ or ‘other illicit substances’ is made explicit by study authors. Total number of recurrences can include more than one type of substance. We distinguish between substance relapse during and after treatment.

Predictors of these outcomes include but are not limited to demographic characteristics, historical characteristics such as history of offending, previous hospitalizations and history of substance use, and behavioural characteristics: treatment engagement, incidents of aggression in care, etc.

#### 2.1.5 Study designs

The following study designs were included: experimental studies such as randomised controlled trials and non-randomised controlled studies; non-experimental studies such as cohort studies, cross-sectional studies and case-studies. The following article types were included: peer reviewed articles, doctoral dissertations, and book chapters reporting empirical data. The following articles were excluded: introductions to special editions, book reviews, obituaries and literature reviews (primary sources were sought).

### 2.2 Data extraction and study selection

Key study data were extracted and recorded in a spreadsheet. These data include: authors, location of study, journal, year, type of study design, participants, description of secure setting (e.g., level of security), sample size, type of intervention if any, outcome variables, predictor variables, method of data analysis and the study findings. Data were extracted by JT, EM, PW and JW.

**Figure 1:**
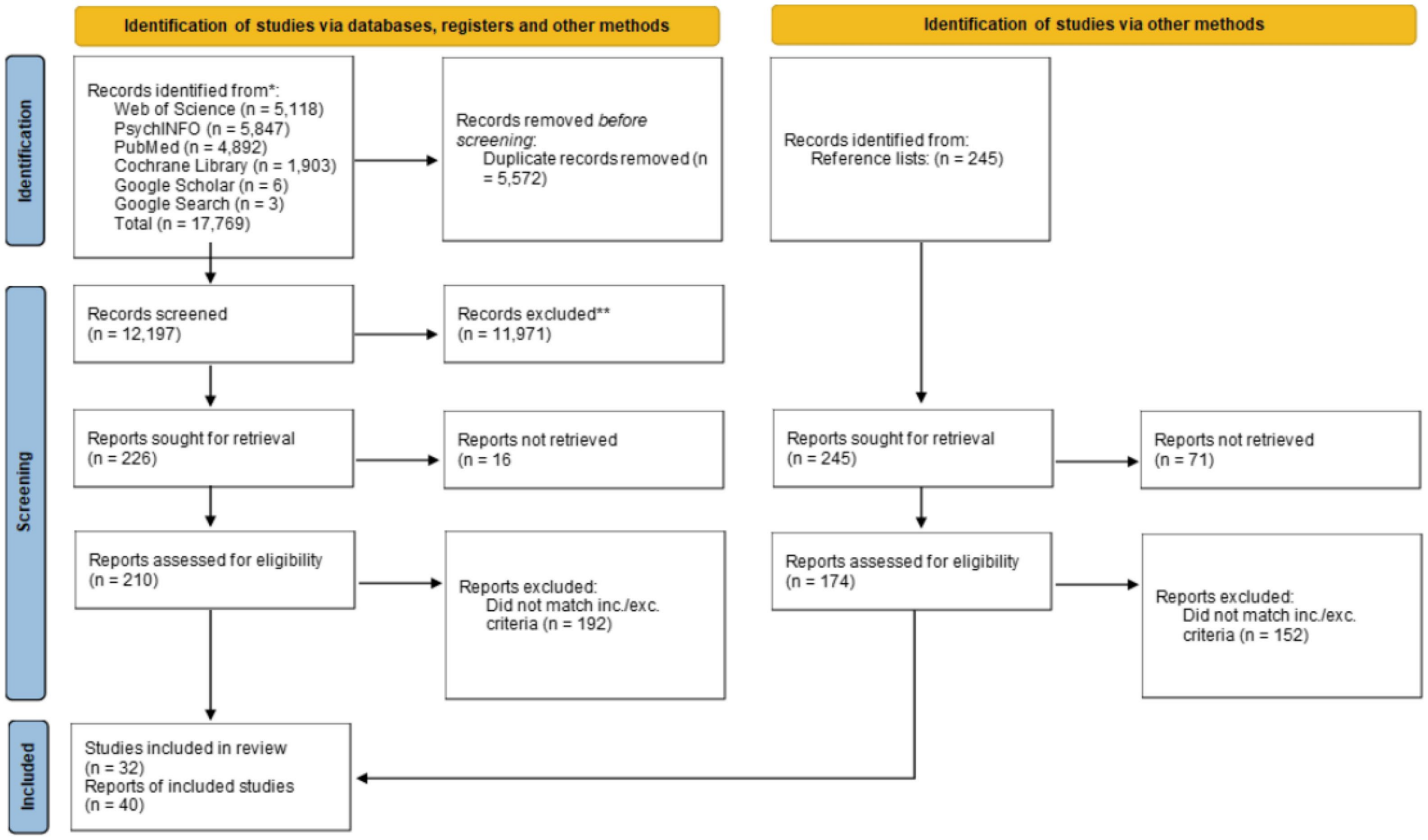
PRISMA flow diagram for initial search (updated search reported in section 2.2).

Our initial search returned 17,769 results (see PRISMA flow diagram in Figure 1); 5,572 results were removed after de-duplication. The titles and abstracts of 12,197 results were screened, with 11,971 removed; 226 results were sought for retrieval; 16 of these could not be found due to lack of access (authors were contacted where contact details were available). The results of 210 publications were assessed for eligibility and 192 of these were excluded in line with the review inclusion and exclusion criteria, leaving 18 hits. The reference lists of these 18 remaining papers were screened and 245 possible additional results were identified, 71 of these could not be accessed due to lack of access. Of these additional results, 174 were assessed for eligibility, of which 152 were excluded according to the review inclusion and exclusion criteria, leaving 23 hits. This left a total of 40 publications reporting on 32 studies. An updated search returned 23 possible hits for full text screening after deduplication. Five of these additional papers were added following eligibility screening.

Publications included in the review are indicated with an asterisk (*) in the reference list.

Most publications reported on studies with cohort designs, of which 13 were retrospective and 21 prospective; there were six publications reporting cross-sectional studies; two retrospective case-controlled studies; and one case study. Sample sizes ranged from *N* = 2 (24) to *N* = 2,483 (25). Date of publication ranged from 1988 to 2023. There was a clear increase in the quality of reporting in the later studies rendering data extraction in more recent publications much easier. Five publications were in the English language, the rest in German.

### 2.3 Data synthesis

Meta-analysis would not have produced meaningful insights given the number of variables measured in the literature and heterogeneous operationalisations [for example, number of past criminal offences was variously measured as: low, medium, and high severity in Gericke and Kallert (26); total number of officially recorded convictions in, for example, Querengässer et al. (27); and number of self-reported offences in Butz (28)]. Therefore, we have chosen to present the findings as a narrative synthesis in line with the general framework proposed by the Centre for Reviews and Dissemination (29). We do not articulate a theory of change as the treatment programmes, settings, and organisational cultures of the different forensic psychiatric hospitals that deliver mandatory treatment for this population over the studied time period vary widely. We develop a preliminary synthesis in the tables and text presented in our results section. We explore the relationships between studies by commenting on whether studies that investigate relationships between the same or similar variables draw convergent or divergent conclusions. We finally assess the robustness of our synthesis in the discussion section.

To strengthen the findings of the review, the sections below are a synthesis of findings relating to predictors that were investigated in more than one study. Significant and non-significant findings are reported. We present the findings relating to each primary outcome thematically (with the exception of the recurrence of substance use, for which there were fewer studies, and we present these findings in one section): legal and criminological; treatment and mental health; and social and demographic categories. Median values were the preferred measure of central tendency given the heterogeneity in outcome variables such as sample sizes and follow-up periods. Only publications reporting on studies using a cohort or cross-sectional design are included in the summary tables to increase the generalisability of findings. Case studies or studies of interventions are not included in these tables or used to contribute to median primary outcome values (e.g., reconviction rates); the findings from these studies are included in the textual description of results.

## 3 Results

### 3.1 Reconviction/reoffending

#### 3.1.1 Summary

Seven studies reported *reconviction rates after discharge*. Table 1 shows the number of patients who were reconvicted for an offence during a median follow-up of 30 months. Median and interquartile range values are reported for these officially recorded offences. The median reconviction rate was 41% over these follow-up periods. Seven studies reported *reoffending rates after discharge*. Four studies reported *reoffending during treatment*; see Table 2. Follow-up periods, definitions of recidivism, and sample sizes varied substantially across studies.

**Table 1:** Rates of reoffending/reconviction after discharge.

Reconviction after discharge			
Study	Reconviction %	Follow-up length	Sample size N
Koch (30)	70%	Average 30 months	63
Dessecker (31)	41%	24 months	249
Gericke and Kallert (26)	36.7%	24 months	120
Bezzel (32)	35.4%	12 months	173
Passow et al. (33)	42.86%	36–48 months	84
Butz (28)	25.1%	Average 40.2 months	251
Querengässer et al. (27)	66%	36 months	261
Median	41%	30	173
Q1, Q3	36.1, 54.4	24, 39.6	102, 250
Other reports of reoffending after discharge			
Leygraf (34)	37%	48 months	136
Pfaff (35)	37%	Average 25.3 months	41
Bezzel (36)	19.1%	Average 14.4 months	136
Hartl (37)	15.5%	12 months	76
Bezzel (38)	16%	12 months	102
Dudeck et al. (39)	17%	12 months	62
Franke et al. (40)	14%	12 months	272

**Table 2:** Other (non-official) reports of offending during treatment.

Study	Reoffending %	Sample size N
Schalast (41)	24.3%	136
Schalast (42, 43)	8.75%	80
Hartl (37)	6.1%	76
Bezzel (32)	6.9%	805

#### 3.1.2 Legal and criminological

Studies investigating the relationship between the number of previous convictions and reconviction mostly found that patients with lengthier criminal records reoffended more frequently (26–28). A study by Querengässer et al. (20) using the same sample as Querengässer et al. (44) found a significant relationship between number of past convictions and reconviction for patients who were transferred from forensic care to prison (which remained significant when included in a multivariate regression model) but not for patients discharged directly into the community. Butz (28) found that rates of reconviction were significantly positively correlated with substance-related index offences, and that having no prior offence was linked with lower rates of reconviction. Querengässer et al. (27) found a range of offences positively correlated with reconviction rates with the strength of the relationship in the following descending order: property crime, assault, an ‘other’ category including traffic offences, arson, ‘other’ violent crimes including attempted offences, homicide, offences against the Betäubungsmittelgesetz (BtMG; Narcotics Law), and sexual offences. Koch (30) reported no significant association between index offence type and reconviction, a finding replicated in Bezzel (32) study of reoffending. No consistent statement can be made on likelihood of reconviction given offence type.

#### 3.1.3 Treatment, mental and physical health

There was mixed evidence for the link between the recurrence of substance use during treatment and reconvictions. Gericke and Kallert (26) found that patients who reused substances during treatment committed a greater number of convicted offences at follow-up; conversely, Butz (28) reported that patients who reused substances less frequently had a greater number of convicted offences, and Koch (30) reported a non-significant relationship. Two studies reported that the recurrence of substance use after discharge was linked to higher reoffending (35, 36). ADHD symptoms were not significantly associated with number of reconvictions after discharge; however, ADHD symptoms were linked with a greater risk of receiving prison sentence after discharge (45).

#### 3.1.4 Social and demographic

Patients who completed job training prior to their current treatment stay were generally less likely to have been reconvicted at follow-up than those not completing job training. However, evidence for this was mixed. While Butz (28) and Querengässer et al. (27) found significant negative associations with reconviction, this was not found in an earlier study by Gericke and Kallert (26) who reported a non-significant relationship. In fact, a subsequent publication from Querengässer (46) divided the sample into successful and unsuccessful treatment completers and found that this significant relationship only held for patients in the unsuccessful treatment group (however, it ceased to be a significant predictor in this group when included in a multivariate regression).

Age at admission was negatively linked with reconvictions in Butz (28), but Gericke and Kallert (26) reported a non-significant finding. Interestingly, Querengässer (44) found a significant negative association for patients transferred to prison and then released (in both univariate and multivariate regressions), but not for patients discharged directly into the community. A similar pattern distinguished these studies on the variable ‘growing up in care’, with Butz (28) finding that this was associated with reconviction, while Gericke and Kallert (26) again reported a null finding. Franke et al. (40) reported no significant difference in reoffending after discharge between men and women.

There was mixed evidence regarding the protective role of intimate relationships and social supports. Hartl (37) reported that living alone at the time of the offence was predictive of reconviction; here again, Gericke and Kallert (26) present a null finding. Hartl (37) reported that patients who were married or widowed were reconvicted less frequently than patients who were not. In a univariate analysis, Querengaesser et al. (44) also reported that marriage or being a widow reduced the likelihood of reconviction for patients who were discharged from hospital directly to prison; a significant association between relationship status and reconviction was not found for patients who were discharged directly into the community. The univariate relationship found by Querengaesser et al. (44) was not replicated when controlling for other variables. Franke et al. (40) found no link between reoffending and living or work situation. In relation to reoffending (as opposed to reconviction), no significant associations were identified for family status at the time of the index offence in studies by Pfaff (35) and Koch (30).

### 3.2 Treatment completion

#### 3.2.1 Summary

Successful treatment outcome was commonly reported across the included studies, with 22 publications addressing this. See section 2.1.4 for a definition of ‘treatment completion’.

As can be seen in Table 3, the median percentage of patients achieving positive and negative treatment outcomes were similar across studies (e.g., 45 and 47%, respectively). However, patients were more likely to have a negative treatment outcome. Given the varied operationalisations of treatment ‘success’ and ‘failure’ and sampling methods in the studies, caution should be applied when interpreting these overall findings.

**Table 3:** Treatment outcomes.

Study	Positive^outcome^	Negative^outcome^	Other^1^	Sample size N
Schalast (41) and Leygraf (34)	73.6%	16.2%	10.2%	136
Berger et al. (47)	53%	47%	–	103
Schalast et al. (48)*	36.6%	46.7%	16.7%	125
Gericke and Kallert (26)	52.3%	47.7%	–	277
Bezzel (32)	33.3%	49.5%	17.2%	805
Schalast et al. (49–51)*	50%	48.7%	1.3%	149
Kemper (52) phase 2 (2005 cohort)	27%	67%	6%	280
Hartl (37)	43.2%	53.3%	3.5%	994
Rotermund et al. (53)	53.85%	46.15%	–	91
Schalast et al. (54)*	23.43%	69.7%	6.87%	175
Hartl et al. (55)	50.5%	49.5%	–	580
Querengässer et al. (56)	38.6%	61.4%	–	777
Schalast et al. (57)*	47.3%	48.57%	4.13%	315
Rosch et al. (58)	24.6%	35.9%	39.5%	357
Ross et al. (59)	45.1%	54.9%	–	1,467
Franke et al. (40)	38.3%	–	61.7%^2^	501
Berthold (17)	61.9%	38.1%		328
Lutz et al. (60)	63%	34%	–	1884
Reiners et al. (25)^3^	15%	18%	–	2,483
Median	45.1%	47.7%	1.3	328
Q1, Q3	35.0, 52.65%	37, 51.4%	0, 8.5%	162, 791

*Essener Evaluation study. ^1^Other, Death or still being in treatment at time of data collection. ^2^Described as the ‘possibility of parole’. ^3^Not all patients were discharged over the time period studied (i.e., 2018). Only cohort or cross-sectional studies are included in this table. Other studies of, for example, a small number of patients following an intervention where treatment outcome was assessed are not included (these include: Seibold (61), *N* = 16; Querengässer (46), *N* = 12; Fontao et al. (62); *N* = 17). ‘–’ values included in median calculations as ‘0’.

#### 3.2.2 Legal and criminological

The number of previous criminal convictions was clearly linked with treatment completion, with seven studies finding that patients who were successfully discharged from care had significantly fewer past convictions (26, 32, 47, 48, 51, 56, 57). Three studies found that patients who had committed substance-related index offences were more likely to have a successful treatment outcome than patients with violent or property-related index offences (17, 37, 56). Conversely, Kemper (52) found that patients (*n* = 280) with violent index offences were less likely to have a successful outcome – a finding that remained significant in a subsequent multivariate analysis, though this finding was not reproduced in phase 2 of this study. Three studies reported that longer parallel sentences (prison sentences ordered to run alongside treatment) were associated with treatment completion (32, 56, 57); while two found no evidence for this (48, 51).

#### 3.2.3 Treatment, mental and physical health

Links between adverse events, substance use and psychopathy with treatment completion were explored. Patients who completed treatment successfully had significantly fewer escapes/absconding events during treatment (26, 32, 37, 56). Three studies found that patients who did not reuse substances during treatment were more likely to successfully compete treatment (32, 37, 56), but two studies reported null findings (26, 48). While three studies found no evidence that patients whose main substance used was alcohol were more likely to successfully complete treatment [(26, 32, 52), phase 2]; one study did report this finding (47). Kemper (52) found that patients with a personality disorder were significantly more likely to have an ‘irregular’ or unsuccessful treatment outcome, though this finding was not replicated in phase 2 of this study, and Berthold and Riedemann (17) also found no link with personality disorder. Relatedly, Berger (63) reported that psychopathy scores were higher in patients who did not complete treatment. No significant difference in successful treatment outcomes was found when comparing hospitals that offered opioid agonist treatment (OAT) and those that did not; though a significantly higher rate of treatment termination without success was observed in hospitals offering OAT (25). No difference was found for patients receiving/not receiving substitution treatment in Berthold and Riedemann (17). Treatment outcome was not linked to presence of ADHD symptoms [(45, 48, 51, 54, 57); with the exception of two findings in which hyperactivity and inattentiveness were linked in (48), and attentiveness in (51)].

Several studies measured treatment motivation. As different operationalisations were used to investigate this, second-order knowledge claims should be made with caution; however, the cumulative results do implicate an important role for treatment motivation for treatment completion. Schalast (43) found an association between a composite outcome variable (called a ‘problem coefficient’, comprising: reoffending, escape, and the recurrence of substance use) and motivational disposition measured across several variables such as resolve to stay abstinent, hope for therapy, and cooperativeness. Using a questionnaire-based measure of therapeutic alliance with a subdomain of ‘engagement’, Fontao et al. (62) found significant differences for successful and unsuccessful completers, with higher engagement scores in the former group. Querengässer et al. (64) identified ambiguous treatment motivation as one of several reasons given for treatment failure in a retrospective cross-sectional study. Treatment motivation was assessed by the authors by looking for evidence of motivation in statements written by therapists, a method the authors describe as subjective and open to interpretation. Using latent class analysis and regression methods, Rosch et al. (58) developed three groups of offenders: the group for whom treatment completion was most likely was characterised, among other things, by higher treatment motivation. Passow et al. (33) found that patients who were motivated to stay abstinent reoffended less frequently after discharge than their peers. Querengässer et al. (46) found a non-significant link with treatment completion but had a sample of only *N* = 12.

#### 3.2.4 Social and demographic

Educational and vocational variables were examined, with evidence of association with treatment outcomes mixed. Patients with successful treatment outcomes were more likely to have completed job training in three studies (37, 47, 61), but five studies reported that this relationship was non-significant (17, 26, 48, 51, 57). Three studies found that treatment completion was associated with having obtained a high school degree (32, 37, 47); while three studies led by Schalast found this not to be the case (48, 51, 57). Patients who were employed prior to treatment were more likely to complete treatment successfully in three studies (48, 51, 56); however, one study did not report this: Schalast et al. (57). Men were more likely to have a negative treatment outcome in Franke et al. (40) and Hartl (37); though no significant difference was observed in Berthold and Riedemann (17). Patients with a migration background were less likely to be successfully discharged from services (60). Berthold and Riedemann (17) found no link between age and treatment outcome.

Living with a partner before entering treatment was linked with positive treatment outcomes in two studies (32, 37); but not in the study by Gericke and Kallert (26). Three studies found that patients who were not brought up in care were more likely than their peers who were brought up in care to complete treatment successfully (26, 48, 51); one study reported null findings in relation to this factor (57).

### 3.3 The recurrence of substance use

#### 3.3.1 Summary

Eleven studies investigated *the recurrence of substance use during treatment*; see Table 4. The sample sizes in these studies ranged from *N* = 39 to *N* = 805, with the ‘follow-up’ period defined as the length of a patient’s inpatient treatment which varied from 12 to 24 months. On average across these studies, slightly over half of the patients reused, with reuse rates ranging from 16.7 to 81.6%.

**Table 4:** Rates of the recurrence of substance use during treatment.

Study	Reuse %	Sample size N
Koch (30)	38.1%	97
Schalast (41)	81.6%	136
Dessecker (31)	79%	249
Seifert and Leygraf (65)	29.9%	144
Schalast (43)	55%	80
Schalast et al. (48)	17.3%	125
Gericke and Kallert (26)	45.85%	277
Bezzel (32)	55%	805
Berger (63)	16.7%	102
Hartl (37)	54.5%	76
Querengässer et al. (64)	64.1%	39

Nine studies reported *the recurrence of substance use after discharge*; see Table 5. Sample sizes in these studies ranged from *N* = 41 to *N* = 501. Not all studies reported average follow-up periods, but these ranged from 12 to 42 months after discharge. The rate of substance reuse across these studies ranged from 34.9 to 85.4%.

**Table 5:** Rates of the recurrence of substance use after discharge.

Study	Reuse %	Sample size N	Follow-up period
Koch (30)	83.8%	63	Average 30 months
Dessecker (31)	69%	249	24 months
Pfaff (35)	63%	41	Average 24 months
Bezzel (32)	47.9%	173	12 months
Bezzel (36)	34.9%	136	Average 14.2 months
Hartl (37)	59%	76	12 months
Passow et al. (33)	85.4%	84	36–48 months
Dudeck et al. (39)	44%	62	12 months
Franke et al. (40)	39%	501	12 months

Six studies examined factors associated with the recurrence of substance use during or after discharge. Fifteen such factors were investigated with only one being included in more than one study. Two studies reported that patients whose main substance use problem was alcohol use were less likely to reuse substances during treatment compared to patients for whom their main substance use problem was defined as any other substance (32, 37). Schalast (43) confirmed these findings when investigating patients at a six-month follow-up, but reported that this distinction was no longer significant at 12 months.

## 4 Discussion

This review synthesised the literature describing studies of the mandatory substance treatment patient population in Germany (§64 German Penal Code) in relation to three primary outcomes: reconviction/reoffending, treatment completion, and the recurrence of substance use. In many ways, our review reports similar findings to the German language reviews by Fries (21) and Querengässer and Baur (22, 23). The general picture suggests that certain risk factors for reconviction/reoffending identified in the general offender population (e.g., greater number of past convictions, lower age at conviction) were also associated with reconviction/reoffending in this population but that very few clear patterns emerged across all studies; that dynamic factors, such as higher levels of treatment motivation, are important predictors of treatment completion, but are under-researched compared to static and historic factors; and that the evidence-base for this population is mixed, often due to heterogeneity in research methods/construct operationalisation. The take-home messages from this review and suggestions for future research are discussed below.

### 4.1 Reconviction and reoffending

On average, reconviction rates (Mdn = 41% over Mdn = 30 months) were higher than those reported for forensic patients in Germany with major mental disorders [receiving treatment under §63 German Penal Code; e.g., 35.2% across a mean follow-up of 16.5 years reported in Seifert et al. (66)]. Franke et al. (40) directly compared the reconviction/reoffending rates of these two groups in a single federal state and confirmed higher recidivism rates in patients with substance use disorders (SUD). The rates found in our review are also higher than reconviction rates for non-forensic patients leaving prison settings in Germany: approximately 34% of people leaving prisons reoffend within 3 years (67). While indicative of the outcomes for the forensic SUD population, these groups are not entirely comparable as individuals have not been matched on factors like age, index offence type, and gender as part of a single prospective study.

Our finding that predictors of reoffending differed in some ways for successful and unsuccessful completers (e.g., number of past convictions, completed job training, age, marital status) might reflect wider national debates about the appropriateness of the §64 German Penal Code treatment order for a large number of people (19). A higher number of past convictions, a lack of job training completion prior to their current forensic treatment, a lower age at admission, and being single were significantly positively correlated with reconviction/offending for patients transferred to prison; while no significant associations were reported between these variables and reconviction/offending for patients transferred to the community. The unsuccessful completers might more closely resemble general (i.e., non-forensic) population offenders, for whom most models predicting reoffending have been developed and thus explain why some predictors were more predictive of reconviction in this group.

### 4.2 Treatment outcome

Across 22 publications in our review, patients were on average more likely to have a negative treatment outcome than a positive treatment outcome (47 vs. 45% respectively; though note the limitations section when considering this median value). This pattern is repeated in several of the largest studies. The importance of this is compounded by studies demonstrating that unsuccessful completers reoffend at higher rates than patients completing treatment successfully (40). One possible explanatory factor here might be *systemic,* that courts are sentencing people to mandatory SUD treatment based on incorrect assumptions about the helpfulness of treatment or broad interpretations of the §64 StGB treatment order by courts or psychiatric experts (40). A second factor might point to the *absence of readiness/motivation* as an important predictor of engagement and success. Motivation has been identified as a key antecedent to positive outcomes in substance use programme participation and recidivism in the international literature (12, 14, 68); in our review (see section 3.2.3); and is viewed by staff as an important factor in treatment outcome in this population (69, 70). A third factor might be *methodological:* treatment effects could be moderated by evaluation methods, program features, and treatment contexts.

A recent study highlights the possible role of demographics in the effectiveness of treatment. Berthold and Riedemann (17) used data collected in a large national survey of 2046 §64 StGB patients. A multivariate regression (*N* = 326) indicated that three variables were significant predictors of treatment completion: being in work in the 6 months prior to the index offence and intoxication at time of the index offence were associated with greater odds of treatment completion; having a migration background was associated with greater odds of treatment failure.

Explaining this latter finding, the authors propose that treatment engagement might by affected by lack of knowledge of the German language and provision of support in a range of languages, not migration background *per se*. Ross et al. (71) note that forensic patients with a migration background in Germany face a range of factors that complicate their health and social needs, including socioeconomic status, racism, access to education, fleeing from war or persecution, factors linked to precarious travel from home countries, and the cultural and linguistic adaptations to life in Germany. These factors are linked to challenges in assessment and treatment engagement (72). It should be noted that Kemper (52) found no significant link between either nationality or language difficulties and treatment outcome, however. The regression in Berthold & Riedemann (17) accounted for 24% of outcome variance, thus leaving considerable room for further explanatory factors. The only variables consistently associated with successful treatment completion across multiple studies in our review was the occurrence of escapes/absconding events and number of past convictions (successful treatment completers had significantly fewer escapes/absconding events and past convictions).

### 4.3 Recurrence of substance use

Studies of the recurrence of substance use included in our review found that approximately half of all patients reused substances during treatment, and around 60% reused after discharge, with follow-up periods ranging from 12 to 48 months. Reviews have highlighted high substance use recurrence rates across a range of substances in the general population during and after treatment (73). These recurrences can occur soon after initiating treatment, with several studies reporting recurrence of substance use within weeks to months in two-thirds of some study participants (73). A comparative study of outcomes for (a) criminal justice-mandated patients (*n* = 141), (b) justice-involved, non-mandated patients (*n* = 235), and (c) patients not involved in the CJS following substance use treatment in the US (*n* = 1,719) found that 53.9, 45.3 and 39.9% of people in these groups, respectively, remained abstinent after 1 year (74). Higher abstinence rates for the criminal justice-mandated patients were not explained by before-treatment differences.

### 4.4 Robustness of the synthesis and directions for future research

Overall, the findings of our review suggest that although a large number of studies have been conducted to identify the predictors of successful treatment and criminological outcomes for this population, there remain three problem areas. The first is *methodological*; although many studies in our review tested the same relationships between x and y variables (e.g., vocational training and reconviction), the operationalisations of these varied widely, particularly at the predictor level. This renders meta-analysis unsuitable. The second is *practical*; most variables are static and historical as these are easier to routinely collect. This neglects important dynamic treatment and risk factors that are known to be important for predicting outcomes (75–77). The third is *conceptual*; study authors [with a few exceptions, e.g., speaking patterns in a therapeutic programme as an indicator of engagement in Querengässer (46)] did not choose variables/factors that fall firmly within recent developments in offender rehabilitation, namely strength- and recovery-based approaches, or risk assessment that incorporates protective factors or shared risk assessment. None of the included studies explicitly measured domains of personal recovery, protective factors, fundamental goods as identified in the Good Lives Model (78), or patient participation in treatment, for example in shared risk assessment (79). A growing body of literature supports the claim that strength- and recovery-based models of treatment are valuable concomitants to medical, risk, or deficit-based approaches (80).

Researchers should therefore agree upon variable operationalisations and methods of data imputation and collection. This can be achieved by the widespread adoption, expansion and harmonisation of pre-existing surveys and databases. Berthold and Riedemann (17, 81) annually collect data from across Germany: the ‘§64 StGB Stichtagserhebung’ (‘cut-off date survey’). A nationwide effort should be undertaken to adopt this in all forensic hospitals with forensic patients receiving §64 StGB care for SUDs. In furtherance to this nation-wide survey/database, a working group should be established to choose routinely used measures of strength- and recovery-based treatment. Given the relevance of therapeutic milieu and the efficacy of therapeutic community (TC) approaches, measures of ward atmosphere would also be beneficial (82). Further, researchers should ensure they consider gender differences in outcomes as some recent research of this population found that differences in outcomes across sex can be observed: a similar proportion of men (55%) and women (55%) completed treatment, whereas men (42%) were more likely to be released to prison than women (29%); rehospitalisation was significantly linked to homelessness and unemployment in women but not men (40).

### 4.5 Limitations

Several limitations of this review should be acknowledged. First, literature for this review was sought by using search terms in the English language. This returned a reasonable number of relevant hits. However, the number of eligible publications found following hand searches of the reference lists of the initially returned hits suggests that (A) the initial search should have been conducted in the German language too, but (B) the low number of relevant results was compensated for as 245 potentially relevant publications were subject to full-text review and 26 publications were included. Second, it was not always possible to discern how authors operationalised certain variables, such as treatment ‘success’ or ‘failure’. We have attempted to be as consistent and clear as possible in reporting results and to inform the reader of these differences across studies; we also chose not to undertake a meta-analysis for this reason. Third, it was not always clear across different studies whether the same or overlapping samples were reported upon. Where this was clearer (e.g., Essener Evaluation Study) we indicated this. However, it is possible some patient data have been reported on more than once. Fourth, median values presented in this review are taken from a range of studies with various sampling methods (e.g., cohort, cross-sectional). Therefore, median values are indicative only and cannot be said to be definitive and generalisable for all patients at all hospitals in Germany. Finally, reoffending and reconviction offence categories and types of substances were not widely reported in studies. Predictors of reoffending, reconviction, and substance use will likely be different for different offence categories and substance types, so this should also be considered in future research.

## 5 Conclusion

Evidence of the effectiveness of mandatory substance use treatment is mixed. Criminal justice-involved persons with substance use disorders in Germany can be ordered to received mandatory treatment in forensic hospitals (§64 German Penal Code). A concerted effort has been undertaken by researchers to identify predictors of recidivism, treatment completion, and the recurrence of substance use in this population. Predictors of recidivism in some ways reflect wider models predicting recidivism (e.g., age), but in many cases evidence was mixed (e.g., job training completion prior to treatment). The explanatory power of models predicting treatment completion and heterogeneity in findings in relation to this outcome suggest that more sophisticated studies using dynamic variables are needed. Much of this body of research suffers from methodological, practical, and conceptual limitations. The appropriateness and ethics of mandatory treatment under §64 German Penal Code for this population are current topics of debate in German forensic mental health.

## Data availability statement

The original contributions presented in the study are included in the article/supplementary material, further inquiries can be directed to the corresponding author.

## Author contributions

BV: article conception, second draft, and editing. JT: article conception, first draft, data extraction and synthesis, and editing. JW: data extraction and synthesis. EM: data extraction and synthesis. All authors contributed to the article and approved the submitted version.
